# Robust and fast characterization of OCT-based optical attenuation using a novel frequency-domain algorithm for brain cancer detection

**DOI:** 10.1038/srep44909

**Published:** 2017-03-22

**Authors:** Wu Yuan, Carmen Kut, Wenxuan Liang, Xingde Li

**Affiliations:** 1 Department of Biomedical Engineering, Johns Hopkins School of Medicine, Baltimore, MD 21205, USA

## Abstract

Cancer is known to alter the local optical properties of tissues. The detection of OCT-based optical attenuation provides a quantitative method to efficiently differentiate cancer from non-cancer tissues. In particular, the intraoperative use of quantitative OCT is able to provide a direct visual guidance in real time for accurate identification of cancer tissues, especially these without any obvious structural layers, such as brain cancer. However, current methods are suboptimal in providing high-speed and accurate OCT attenuation mapping for intraoperative brain cancer detection. In this paper, we report a novel frequency-domain (FD) algorithm to enable robust and fast characterization of optical attenuation as derived from OCT intensity images. The performance of this FD algorithm was compared with traditional fitting methods by analyzing datasets containing images from freshly resected human brain cancer and from a silica phantom acquired by a 1310 *nm* swept-source OCT (SS-OCT) system. With graphics processing unit (GPU)-based CUDA C/C++ implementation, this new attenuation mapping algorithm can offer robust and accurate quantitative interpretation of OCT images in real time during brain surgery.

It is challenging to distinguish cancer from non-cancer tissues in brain cancer surgery, especially at the infiltrative zones. Incomplete resections often lead to disease recurrence and negatively affect survival. Safely maximizing the extent of resection while preserving functional non-cancer tissues is the key to achieve optimal outcome^1^,^2^. Therefore, it is imperative to visualize cancer versus non-cancer tissues during surgery. Technological advances have been made for this end with the goal of providing real time detection of brain cancer intraoperatively, including intraoperative ultrasound, intraoperative computed tomography (iCT), fluorescence-guided surgery, intraoperative magnetic resonance imaging (iMRI), and Raman spectroscopy^3^,^4^,^5^,^6^,^7^,^8^. However, these imaging technologies have various limitations in terms of resolution, field of view, three dimensional detection, quantification, and continuous guidance capability.

As a noninvasive, label-free, and cost-effective technique, intraoperative OCT (iOCT) has attracted increasing interest for surgical guidance because it can provide high-resolution and real-time feedback to surgeons^9^,^10^,^11^,^12^. Currently, iOCT is mainly used for eye surgery, where tissues have distinct structural layers and OCT intensity images with a resolution of 1–10 *μ*m are able to provide direct visual cues for delineating tissues in real time. It is significantly challenging to intraoperatively classify cancer versus non-cancer in brain tissues where there are no any obvious structural landmarks at micron-to-millimeter scale. In this scenario, there is a clear need for interpreting the OCT data not just qualitatively, but also quantitatively based upon the intrinsic tissue optical properties which govern light absorption, scattering and propagation within different tissues.

The optical attenuation coefficient (as defined by the sum of absorption and scattering) is a quantitative parameter that can be extracted from OCT intensity images^13^,^14^,^15^,^16^,^17^,^18^,^19^,^20^,^21^. Recent publications have demonstrated the feasibility of using this parameter for classifying pathological lesions in both *ex vivo* and *in vivo* tissues^14^,^15^,^17^,^18^,^20^,^21^. For example, Cauberg *et al*. used the attenuation coefficients of bladder biopsies to grade urothelial carcinoma^17^. More recently, an encouraging progress has been demonstrated by Kut *et al*. to differentiate infiltrated human brain cancer from non-cancer brain tissues by using a real-time, attenuation mapping method^21^.

For intraoperative detection of brain cancer, a robust, accurate, and high-speed OCT attenuation mapping is needed to improve surgical results. Most current methods used to calculate the optical attenuation coefficient adopt a single-scattering model, which corresponds to a single exponential decay of light intensity in a homogeneous tissue along imaging depth (if not considering the beam focusing effect)^13^,^15^,^16^,^19^,^21^. To quantify the optical attenuation coefficient, the following fitting methods, which will be detailed in the Methods Section, can be used: (1) the exponential fitting method (EF)^13^, which is robust but time-consuming as it involves an iterative procedure; or (2) a log-and-fitting method (LF)^15^,^16^,^19^,^21^ which is fast but prone to the detrimental effects of few-scattering, especially in heterogeneous and/or highly scattering tissue^22^. Furthermore, it is found that both EF and LF methods are sensitive to the position of tissue surface and become less accurate if the tissue surface is not flat and/or static, while during brain surgery the tissue surface often demonstrates complex geometry and motions caused by various physiological and/or environmental movements. As a result, a new robust and fast attenuation characterization method is needed for successful translation of this optical property mapping technology into intraoperative use.

In this paper, we report a new generic frequency-domain (FD) method which uses Fourier transforms to enable robust, accurate, and fast characterization of the optical attenuation coefficient. Firstly and most importantly, since both EF and LF methods require an accurate detection of the tissue surface from the OCT signals, we found the FD method is very robust in characterizing OCT signals even with incorrect surface detection, while with EF and LF methods this will produce inaccurate attenuation coefficients. This robustness is especially important in *in vivo* surgical applications. Furthermore, when compared with the LF method, the FD method is less susceptible to the few-scattering effects without sacrificing its high computational speed. Similarly, compared with the EF method, this new FD method provides significant improvements on computational speed without compromising accuracy. Last but not the least, our FD method is equally robust against speckle noise as the EF and LF methods. In the following sections, we describe the methodology in detail and demonstrate the feasibility results of this new generic FD method, which was validated with both phantom and brain cancer tissue images acquired with a 1310 *nm* SS-OCT system. With GPU-based CUDA C/C++ implementation, our algorithm can be applied to high-speed and accurate optical attenuation mapping in real time with superb robustness, which is imperative for visualizing brain cancer infiltration during surgery.

## Methods

### FD Algorithm

It is well known that the depth-dependent OCT intensity signal *I*_*oct*_(*z*) can be written as:^22^,^23^,^24^


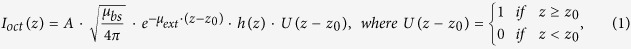


where *z* is the imaging depth, *z*_0_ is the surface of the sample, *A* is an overall system constant, *μ*_*bs*_ is the back-scattering coefficient, *μ*_*ext*_ is the total optical attenuation (extinction) coefficient, and *h(z*) is the point spread function describing the focusing/de-focusing effect of the beam in a turbid medium (*e.g.*, biological tissue).

For accurate assessment of the attenuation properties, we developed a method to mitigate the influence of the depth-dependent effects of the beam profiles as described previously^19^, where the OCT signal from the sample of interest *I*_*s*_(*z*) was normalized with the one from a reference silica phantom *I*_*r*_(*z*) with a known attenuation coefficient (see Supplementary Information). When assuming the same *z*_0_ = 0 for both OCT signals, the following depth-dependent function (termed as normalized OCT intensity) with a single exponential decay starting from the sample surface can be obtained:


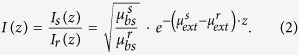


After taking the Fourier transform of equation (2) and some algebra, the optical attenuation coefficient can be derived by comparing the amplitude of any two harmonic coefficients in the Fourier domain, such as by taking the ratio of the direct current (DC) component with the modulus of the first harmonic coefficient, *i.e.*:


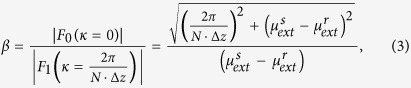


where κ is the frequency in space, Δ*z* is the pixel size along depth, and *N* is the total number of data points (*i.e.*, pixels) per A-line. This FD method was inspired by the frequency domain method for computing fluorescence lifetime^25^. For a given discrete A-line data, *i.e., I(n*·Δ*z*), the amplitude of any harmonic coefficients (denoted by the order *m*) is computed by the following discrete Fourier transform (which is intrinsically extremely fast):


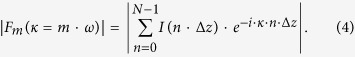


Here 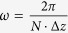 is the fundamental spatial frequency, and *n* = 0 to *N* − 1 represents the sequential index of the given discrete A-line. To summarize, we can use this new FD method and readily apply discrete Fourier transform to the discrete A-line data to calculate the amplitudes of any two harmonics, obtain the relative attenuation coefficient term, *i.e.*, 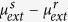, and finally, extract the optical attenuation coefficient of the sample, *i.e.*, 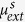, given the known attenuation of the reference phantom 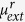.

Alternatively, an iterative exponential fitting process with a trust-region algorithm^26^, *i.e.*, the traditional EF method, can be applied to equation (2) to extract the relative attenuation coefficient 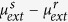 from which the sample attenuation coefficient 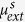 can be readily obtained^13^. LF method, on the other hand, uses the logarithm of equation (2) to achieve a linear equation and then 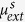 can be derived from the slope of the linear equation by using the simple linear regression algorithm (linear least squares method, see Supplementary Information)^15^,^16^,^19^,^21^.

### SS-OCT System

In order to quantitatively evaluate the ultrafast FD method, an SS-OCT system was used to acquire datasets from the reference silica phantom and from a freshly resected human brain cancer sample. The SS-OCT system is equipped with a home-built FDML swept source which operates at 220 kHz and a center wavelength of 1310 *nm* with a 3 dB bandwidth of ∼110 *nm* and a full bandwidth of ∼150 *nm*. A handheld probe with an x-y galvanometer was used to perform 2D beam scanning. The power incident on the samples was about 15 *mW*. Measured lateral and axial resolutions are ∼16.0 *μ*m and ∼6.4 *μ*m (in tissue), respectively. A detection sensitivity of ∼126 dB was achieved.

### Images Acquisition and Processing

For both the silica phantom and human brain cancer tissue, we acquired OCT data over a 2 *mm* × 2 *mm* × 1.8 *mm* (x × y × z) volume. This produced three-dimensional datasets with 2048 data points/A-line × 2048 A-lines/frame × 256 frames. To obtain a color-coded attenuation map, we reduced the speckle noise by averaging approximately ∼330 A-lines along the x-direction, with a window step size of ∼13 A-lines. After that, an automatic algorithm was implemented to locate the sample surface for each averaged OCT intensity signal *I*_*s*_(*z*) (tissue and sample phantom) and *I*_*r*_(*z*) (reference phantom). Using the identified sample surface as the starting data point in the z-direction, we compute the optical attenuation coefficient with an appropriate data length (∼350 vs. ∼700 *μ*m) by using the EF, FD, and LF methods. In other words, optical attenuation properties were computed for each voxel of 0.33 *mm* × 0.008 *mm *× 1.8 *mm* (x, y, and z) within the volumetric images with a step size of 0.013 *mm* in the x-direction and 0.008 *mm* in the y-direction.The imaging protocol for human tissues study was approved by the Institutional Review Board at Johns Hopkins University.

## Results

### Comparison of Robustness against the Few-scattering Effect

**Figure 1:**
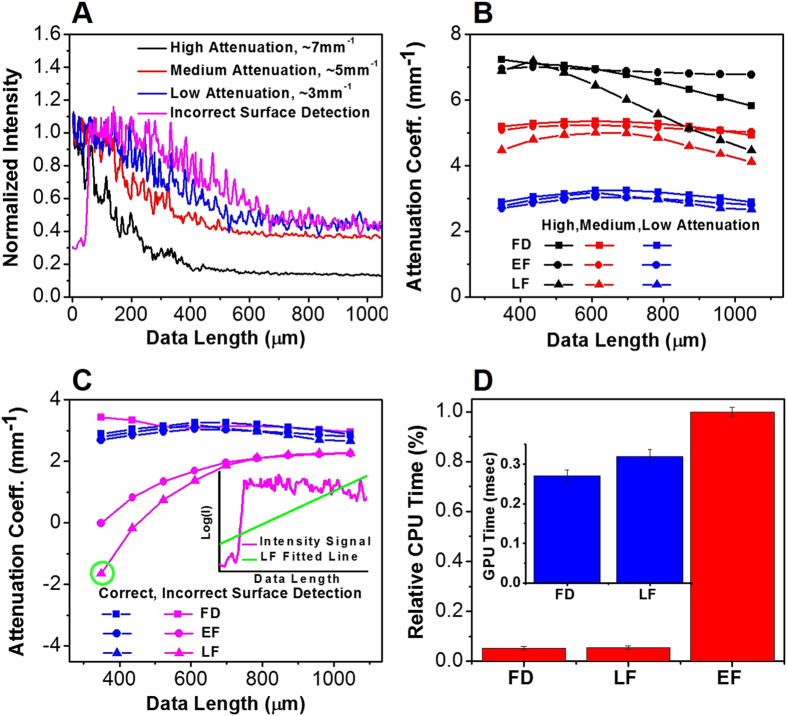
(**A**) Representative normalized average OCT intensity signals *I(z*) with high (black line), medium (red line) and low (blue line) attenuation coefficients. Magenta line shows the same *I(z*) at low attenuation coefficient (blue line), but with incorrect tissue surface detection. These intensity signals were rescaled to a similar range for display purpose. (**B**) and (**C**) Using the same normalized average intensity signals as shown in (**A**), the attenuation coefficients were calculated using the Fourier domain (FD, rectangles), the linear fitting (EF, circles), and exponential fitting (LF, triangles) methods as the data length (in the z-direction) increases. The inset of (**C**) shows the logarithm of OCT intensity signal with incorrect surface detection and a data length of ∼350 *μ*m (magenta line), and the wrongly fitted line using the LF method (green line) which resulted in a negative attenuation coefficient highlighted with the green circle. (**D**) Relative CPU time required to process a dataset with approximately 2048 A-lines/frame × 256 frames using the FD, LF, and EF methods. Inset shows the GPU time (implemented in CUDA C/C++) needed for the FD and LF methods to process one B-frame with 2048 A-lines/frame and 2,048 pixels/A-line.

It is well-known that as light propagates, the probability of few-scattering in a turbid medium increases exponentially with the penetration depth and the attenuation coefficient according to 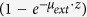
22. While all three methods (FD, LF and EF) are based on a single-scattering model, we know that few-scattering occurs in real-life conditions. Thus, we have evaluated the robustness of all three algorithms against the few-scattering effect, as demonstrated in Fig. 1(A,B).

To conduct this test, we acquired OCT signals from freshly resected human brain tissues and obtained normalized OCT intensity signals at regions with low (∼3 *mm*^−1^), medium (∼5 *mm*^−1^) and high (∼7 *mm*^−1^) attenuation coefficients (as shown in Fig. 1(A)). Notably, 300-A-lines within the same frame was averaged to achieve the OCT intensity signal before the normalization with the corresponding averaged intensity signal from the reference phantom.

Using the same normalized OCT intensity signals, we then evaluate the robustness of all 3 methods as the probability of few-scattering increases. In this test, we increase the probability of few-scattering by 1) increasing the data length (in the z-direction) used to compute the sample attenuation coefficient 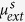; and 2) by using a region of interest with higher attenuation. As shown in Fig. 1(B), the EF method yielded the most consistent computations of attenuation coefficients for all regions of interests and at different data lengths. As expected, the EF method is the most robust against the few-scattering effect, despite its low computational efficiency. Therefore, we treat results from the EF method as the gold standard in subsequent evaluations of the FD and LF methods.

When a region of interest has a low attenuation coefficient at ∼3 *mm*^−1^ (*i.e.*, with low few-scattering effects), we found that FD, LF and EF provided comparable results even when data lengths increased. When the attenuation coefficient increased to the medium level of ∼5 *mm*^−1^ (*i.e.*, with medium few-scattering effects), results from the LF method deviated significantly from the EF method as the data length increases, while the FD method provided comparable results to the EF method. Finally, when the attenuation coefficient is high (*i.e.*, ∼7 *mm*^−1^), the LF method was severely influenced by the few-scattering effect, with ∼35% reduction in the attenuation coefficient at a data length of ∼1050 *μ*m (which corresponds to ∼7.3 mean free paths). In comparison, FD method was only moderately affected by the increasing few-scattering effect (with a ∼ 20% reduction in the attenuation coefficient). Thus, we find that the FD method is more robust against the few-scattering effect when compared with the LF method. This feature can be very important and ensures data reliability when obtaining quantitative attenuation properties.

### Comparison of Robustness against Incorrect Tissue Surface Detection

Then we compared the robustness of all three methods when tissue surface detection is inaccurate. As shown in Fig. 1(A), we used the same imaged region (with 300 A-lines) and calculated the normalized average sample intensity signal *I(z*) when the tissue surface detection is accurate (*i.e.*, the blue line with the low attenuation coefficient at ∼3 *mm*^−1^), and again when the tissue surface detection is inaccurate (*i.e.*, the magenta line as shown in the figure). Here, we intentionally select a region with a low attenuation coefficient for comparison (as a tissue region with high attenuation coefficient already suffers significantly from the few-scattering effect which will mask the true effects of incorrect surface detection).

As seen in Fig. 1(C), when tissue surface detection is inaccurate, both the EF and LF methods exhibited a pronounced deviation from the true attenuation coefficient (∼3 *mm*^−1^). In fact, even negative attenuation coefficients were found when computed using the EF and LF methods. As an example, the incorrect surface detection leaded to a wrongly fitted line with the LF method (green line), as shown in the inset of Fig. 1(C), and resulted in a negative attenuation coefficient (green circle in Fig. 1(C)). In contrast, the FD method is significantly robust against the effects of incorrect tissue surface detection and produced attenuation coefficients which are closer to its true value at ∼3 *mm*^−1^. Such robustness from the FD method is consistent with our theoretical predictions as detailed in Supplementary Information, where we show that an incorrect detection of the sample surface, *i.e., z*_0_, has no effect on our attenuation computations with the FD method. As a result, the FD method is especially desirable for *in vivo* and high-speed applications, where the experimental subject often exhibits complex surface geometry and motions as a result of breathing, heart beat and other bodily movements. Since the FD method eliminates the need for accurate surface detection, it is consequently very useful in minimizing motion errors and achieving clinically meaningful conclusions in real time during a clinical or experimental procedure.

### Comparison of Computational Efficiencies

Furthermore, to quantitatively compare the computational efficiencies of the FD, LF and EF methods, a benchmark test was performed using a phantom dataset with approximately 524,000 A-lines (2048 A-lines/frame × 256 frames, and 2,048 pixels/A-line). Central processing unit (CPU) timing was carried out on a personal computer with a Windows 7 operating system, an Intel Core i5-4570 at a base processor frequency of 3.2 *GHz* and a maximum graphics memory of 2 *GB*. Algorithms were implemented in MATLAB (The MathWorks Inc.). Computational time was measured for all 3 methods. As shown in the benchmark results in Fig. 1(D), we found that the FD method takes 2.38 *msec* per frame and the LF method takes 2.51 *msec* per frame when compared to the EF method which takes 44.92 *msec* per frame. To summarize, the FD method offers a speed slightly better than the LF method, but much faster (approximately 22 times) than the EF method.

To more precisely understand the computational efficiencies of the FD and LF methods in real-time scenarios, we performed an additional benchmark test to study the GPU timing between these two methods. GPU timing was performed using an NVIDIA GeForce GTX 760 with 1152 CUDA cores, a base clock at 0.98 *GHz*, and a memory speed of 6.0 *GB* per second. Algorithms were implemented in CUDA C/C++ using the same personal computer. A dataset of the same size as above was used. As shown in the benchmark results in the inset of Fig. 1(D), the GPU version of the FD method (0.27 *msec* per frame) offers an approximately ∼15% speedup when compared with the LF method (0.32 *msec* per frame). This speedup is likely due to the fact that the FD method relies on CUDA computation of 3 key parameters: 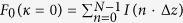, 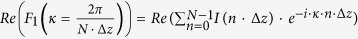, 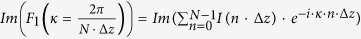 (as shown in equation (4)), while the LF method relies on CUDA computation of 4 key parameters for the simple linear regression (linear least squares) algorithm: 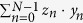, 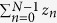, 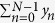, and 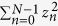 (see Supplementary Information for the explanation of these terms, as shown in the simple linear regression (linear least squares) algorithm). To summarize, the FD method utilizes less shared memory in CUDA, requires fewer CUDA computations, and as a result, enjoys a slightly reduced computational time when compared with the LF method.

Notably, only the computational times for the FD and LF methods were included in the above GPU benchmark test. Times for other supporting algorithms (*e.g.*, loading of OCT intensity data, speckle suppression/data averaging, and surface detection) were identical for both methods, and as a result, were not included in the above analysis.

### Comparison of Robustness against Speckle Noise Levels

Finally, to evaluate the robustness of all 3 methods against speckle noise, we compared the average intensity signals from a region of interest containing 300 A-lines versus 50 A-lines (within the same frame). Our results showed that all 3 methods enjoyed comparable robustness to speckle noise. Using the FD method, there is approximately 8% change in the computed attenuation when averaged with 50 A-lines (versus 300 A-lines). Using the EF and LF methods, there are respectively ∼10% and ∼13% changes in the computed attenuation when averaged with 50 A-lines (versus 300 A-lines).

### Validation of the FD Method with Phantoms

**Figure 2:**
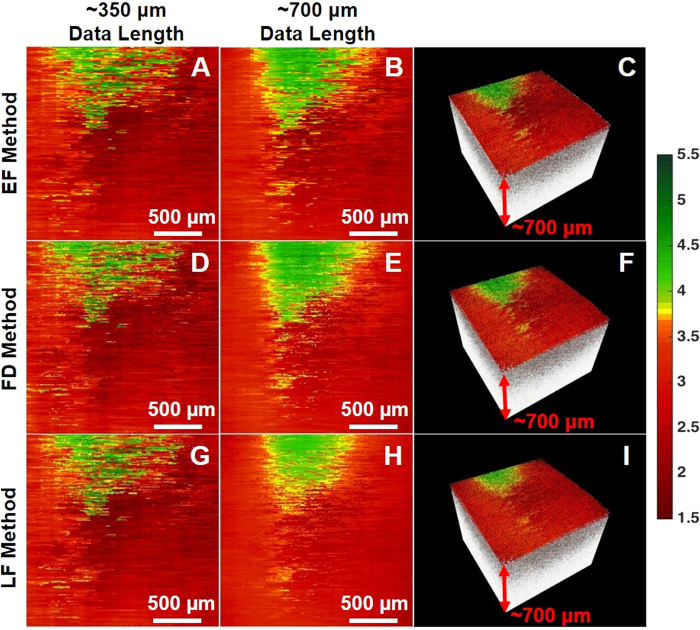
Color-coded OCT attenuation maps calculated for a silica phantom S using (**A** to **C**) the EF method, (**D** to **F**) the FD method, (**G** to **I**) the LF method. The colormaps were obtained by using a data length of (**A**,**D**,**G**) ∼350 *μ*m and (**B**,**C**,**E**,**F**,**H**,**I**) ∼700 *μ*m in the z direction, respectively; each RGB pixel represents the attenuation coefficient with ∼330 A-lines averaged laterally, and a step size of ∼13 A-lines. (**C**,**F**,**I**) The corresponding volumetric OCT image with an overlaid attenuation *en face* colormap. Color bar (units in *mm*^−1^): green represents high attenuation and red represents low attenuation.

We firstly obtained OCT images for two test phantoms: phantom R made with 5% gelatin embedded with 12.5 *mg/mL* silica nanospheres, and phantom S made with 5% gelatin and 50 *mg/mL* silica nanospheres (which is more heterogeneously distributed). Using phantom R as reference and phantom S as our sample, we validated our results by comparing the measured optical properties of phantom S with the analytically calculated values of the same phantom using *Mie* theory. Figure 2 illustrates the *en face* color-coded optical attenuation maps of phantom S obtained with the EF, FD and LF methods with a data length of ∼350 *μ*m. Here, we find that the three methods achieved comparable attenuation maps; if we subtract the colormaps in Fig. 2(A) by the map in Fig. 2(D), the change in attenuation coefficients Δ*μ* is roughly 0.01 ± 0.25 *mm*^−1^, while Δ*μ* is about 0.01 ± 0.18 *mm*^−1^ between the colormaps in Fig. 2(D,G). However, when a longer data length (*i.e.*, ∼700 *μ*m) along the z-direction was used, the change in attenuation Δ*μ* in Fig. 2(E) vs. 2(H) increased to 0.04 ± 0.14 *mm*^−1^, while a relatively small change of 0.01 ± 0.32 *mm*^−1^ in attenuation Δ*μ* was found between Fig. 2(B,E). Most notably, the LF method using the data length of ∼700 *μ*m (Fig. 2(H)) yielded the most inconsistent results, as the green band at the top of the image (signifying high attenuation, with the band boundary determined by a threshold of 3.75 *mm*^−1^) is much smaller and with an ∼35% reduction in width when compared with the other attenuation maps (Fig. 2(A,B,D,E and G)). This suggested that the LF method is more susceptible to the increasing influence of few-scattering effect as the data length increases. In contrast, the FD method produced a relatively robust attenuation coefficient map which is more comparable with that generated by the EF method, regardless of the data length. Finally, the corresponding volumetric OCT images with an overlaid attenuation *en face* colormaps were shown in Fig. 2(C,F and I) for the EF, FD, and LF methods with the data length of ∼700 *μ*m, respectively.

### Validation of the FD Method for Brain Cancer Detection

**Figure 3:**
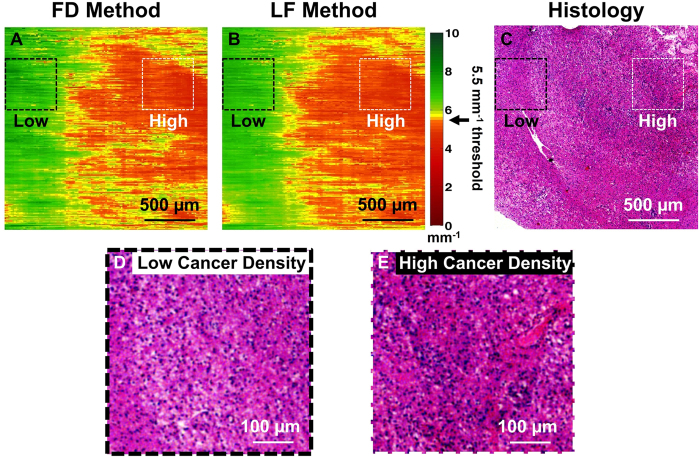
*En face* OCT attenuation colormaps of an *ex vivo*, freshly resected human brain cancer tissue computed using (**A**) the FD method and (**B**) the LF method. The color-coded attenuation map represents areas of low cancer density (indicated by green color, corresponding to high attenuation) and high cancer density (indicated by red color, corresponding to low attenuation). (**C**) Corresponding histology image (H&E stained) of the scanned tissue area for validation. Zoomed-in regions were provided in (**D**) and (**E**) to demonstrate the histology found in areas with low cancer density (black dotted box) and high cancer density (white dotted box), respectively.

To further illustrate the feasibility of our FD method, we obtained a freshly resected human brain cancer tissue sample from a grade IV glioma patient (*i.e.*, glioblastoma). Before imaging, the tissue sample was marked with a yellow margin marking dye (MasterTech) for histological registration and correlation. After imaging, the tissue samples were placed in formalin overnight and then submitted for histological processing. Standard histological slide were obtained and correlated with the attenuation colormaps. Histological interpretation was made independently by a neuro-pathologist who reviewed the histological slides in a blinded study. As shown in Fig. 3, the attenuation colormaps were obtained using the FD and LF methods and subsequently correlated and validated by histology. To obtain the attenuation coefficients, an averaging window of ∼330 A-lines was used with a step size of ∼13 A-lines (along the x-direction), and a data length of ∼350 *μ*m was applied along the z-direction.

Our previous study established an optimal attenuation threshold at 5.5 *mm*^−1^ for distinguishing infiltrative brain cancer margins with high sensitivity and specificity^21^. The same study also found that non-cancer white matter had a significantly higher attenuation when compared with primary brain cancer;^21^ this is because of the high myelin content and lack of necrosis in normal white matter which was well-reported in previous literature^21^,^27^,^28^. Using this diagnostic attenuation threshold, the optical attenuation map of an *ex vivo* human brain sample can be color-coded to clearly show the infiltrative cancer margins. As shown in Fig. 3, low optical attenuation (red) represents areas with high cancer density; medium optical attenuation (yellow) represents areas with medium cancer density; and high optical attenuation (green) represents areas with low cancer density (i.e., the tissue consists of mostly white matter, with diffuse infiltration of some neoplastic components). In the earlier study we have validated the LF method in delineating *ex vivo* human brain cancer versus non-cancer in real time^21^. Our results in Fig. 3 further demonstrate that the FD method is able to achieve comparable results as the LF method in detecting human brain cancer infiltration. Histological validation is provided in Fig. 3(C), with zoomed-in views corresponding to areas of low cancer density (Fig. 3(D)) and areas of high cancer density (Fig. 3(E)). The color-coded optical attenuation map (generated by the FD method) correlates well with histology, which further illustrates the feasibility of the FD method in human brain cancer detection.

## Conclusion

In summary, we developed and validated a new generic, robust, and fast FD algorithm for quantifying tissue attenuation properties from OCT imaging data. Our experimental results demonstrated that the FD method is more robust than both the LF and EF methods when faced with incorrect tissue surface detection. Thus, the FD method is an attractive method which can address this significant real-world problem and provide an accurate attenuation calculation in real time during the surgical procedure. Furthermore, this new method is about 22 times faster than EF method, and is more robust against the effects of few-scattering when compared with the LF method. Our preliminary work shows the potential of this method for robust and fast OCT attenuation imaging/mapping, which is especially desirable for *in vivo* high-speed clinical applications, such as intraoperative brain cancer detection. More research will be carried out to further validate this new method for brain cancer tissue identification and qualification *in vivo*.

## Additional Information

**How to cite this article**: Yuan, W. *et al*. Robust and fast characterization of OCT-based optical attenuation using a novel frequency-domain algorithm for brain cancer detection. *Sci. Rep.* **7**, 44909; doi: 10.1038/srep44909 (2017).

**Publisher's note:** Springer Nature remains neutral with regard to jurisdictional claims in published maps and institutional affiliations.

## Supplementary Material

Supplementary Information
