# Supplementary material for: Robust and fast characterization of OCT-based optical attenuation using a novel frequency-domain algorithm for brain cancer detection
Source: Sci Rep. 2017 Mar 22;7:44909. doi: 10.1038/srep44909 (PMC5361149; doi:10.1038/srep44909)
Supplement: Supplementary Information [file srep44909-s1.pdf]

# Robust and fast characterization of OCT-based optical attenuation using a novel frequency-domain algorithm for brain cancer detection

**Wu Yuan, Carmen Kut, Wenxuan Liang, and Xingde Li***

Department of Biomedical Engineering, Johns Hopkins School of Medicine, Baltimore, MD 21205, USA

* xingde@jhu.edu

## Supplemental information

### Method to Remove the Influence of Depth-dependent Effects of Laser Beam Profiles

In order to extract $\mu_{ext}$, we proposed to first image a reference phantom of known optical properties before imaging the sample of interest. In our experiments, the reference phantom was constructed with home-made mono-dispersive silica nanospheres of $180 \pm 20\ nm$ and a concentration of $12.5\ mg/ml$. Our previous study indicated that the attenuation coefficient of the silica phantom matched very well with predictions by the *Mie* theory. OCT imaging of both the reference phantom and the sample were performed under the same experimental conditions, *i.e.*, with the same incident power, focused spot size, and focusing depth. The corresponding OCT signals for the sample of interest (with a subscript or superscript s) and the reference phantom (with a subscript or superscript r) can be written as:

$$I_s(z) = A \cdot \sqrt{\frac{\mu_{bs}^s}{4\pi}} \cdot e^{-\mu_{ext}^s \cdot (z-z_0)} \cdot h(z) \cdot U(z-z_0), \tag{5}$$

$$I_r(z) = A \cdot \sqrt{\frac{\mu_{bs}^r}{4\pi}} \cdot e^{-\mu_{ext}^r \cdot (z-z_0)} \cdot h(z) \cdot U(z-z_0). \tag{6}$$

Dividing the sample OCT signal $I_s(z)$ in equation (5) by the reference phantom OCT signal $I_r(z)$ in equation (6) and assuming the point spread function $h(z)$ is almost identical for the sample and reference phantoms, we will get the following depth-dependent function with a single exponential decay:

$$I(z) = \frac{I_s(z)}{I_r(z)} = \sqrt{\frac{\mu_{bs}^s}{\mu_{bs}^r}} \cdot e^{-(\mu_{ext}^s - \mu_{ext}^r) \cdot (z-z_0)} \cdot U(z-z_0). \tag{7}$$

### Simple Linear Regression Algorithm in the LF Method

For simplicity, equation (7) can be rewritten as:

$$I(z) = \frac{I_s(z)}{I_r(z)} = C \cdot e^{-\mu \cdot (z-z_0)} \cdot U(z-z_0), \tag{8}$$

where $C = \sqrt{\frac{\mu_{bs}^s}{\mu_{bs}^r}}$ is a constant, $\mu = \mu_{ext}^s - \mu_{ext}^r$ is the attenuation coefficient difference, $z$ is the imaging depth and $z_0$ is the sample surface. When assuming $z_0 = 0$, equation (8) can be further simplified to:

$$I(z) = C \cdot e^{-\mu \cdot z}. \tag{9}$$

The LF method uses the logarithm of equation (9) to obtain a linear equation:

$$\ln(I(z)) = \ln(C) - \mu \cdot z, \tag{10}$$

where $-\mu$ is the slope of the linear equation. For discrete A-line data, *i.e.*, $I(n \cdot \Delta z)$, equation (10) can be rewritten as:

$$y_n = \alpha + \beta \cdot z_n, \tag{11}$$

where $y_n = \ln(I(z_n))$, $\alpha = \ln(C)$, $\beta = -\mu$, $z_n = n \cdot \Delta z$, $n = 0$ to $N-1$ represents the sequential index of the discrete data for a given A-line, $\Delta z$ is the pixel size along the imaging depth, and $N$ is the total number of data points (*i.e.*, pixels) per A-line.

Then $\beta$ can be derived using the simple linear regression (least squares) algorithm, *i.e.*:

$$\beta = \frac{\sum_{n=0}^{N-1} z_n \cdot y_n - \frac{1}{N} \cdot \sum_{n=0}^{N-1} z_n \cdot \sum_{n=0}^{N-1} y_n}{\sum_{n=0}^{N-1} z_n^2 - \frac{1}{N} \cdot (\sum_{n=0}^{N-1} z_n)^2}. \tag{12}$$

### Robustness of the FD Method to Incorrect Surface Detection

To derive $\mu$ with the FD method, we can calculate the Fourier transform of equation (8):

$$F(\kappa) = \int_{-\infty}^{\infty} C \cdot e^{-\mu \cdot (z-z_0)} \cdot e^{-j \cdot \kappa \cdot z} \cdot U(z-z_0) dz = \begin{cases} \frac{C}{\mu + j \cdot \kappa} \cdot e^{-j \cdot \kappa \cdot z_0} & if \quad z_0 \geq 0 \\ \frac{C}{\mu + j \cdot \kappa} \cdot e^{-\mu \cdot |z_0|} & if \quad z_0 < 0 \end{cases}, \tag{13}$$

where $\kappa$ is the frequency in space, $z$ is the imaging depth of OCT signal. Please note that for OCT data analysis, the depth by default cannot be negative. Thus $z \geq 0$ is assumed for deriving the above equation.

Then the optical attenuation coefficient $\mu$ can be derived by comparing the DC component with the modulus of the first harmonic coefficient of equation (13),which leads to:

$$\frac{|F(\kappa=0)|}{\left|F(\kappa=\frac{2\pi}{N\cdot\Delta z})\right|} = \frac{\sqrt{(\frac{2\pi}{N\cdot\Delta z})^2+\mu^2}}{\mu}, \tag{14}$$

where $\Delta z$ is the pixel size along depth and $N$ is the total number of data points (*i.e.*, pixels) per A-line.

Clearly, equation (14) is independent of $z_0$, *i.e.*, the sample surface position; thus in theory the FD method is not sensitive to the accuracy of surface detection.
